# One-dimensional photonic crystal nano-ridge surface emitting lasers epitaxially grown on a standard 300 mm silicon wafer

**DOI:** 10.1038/s41377-025-02061-z

**Published:** 2026-02-24

**Authors:** Eslam M. B. Fahmy, Zhongtao Ouyang, Davide Colucci, Nicolas Le Thomas, Joris Van Campenhout, Bernardette Kunert, Dries Van Thourhout

**Affiliations:** 1 Photonics Research Group, Department of Information Technology (INTEC), Ghent University - imec, Ghent, Belgium; 2 imec, Kapeldreef 75, 3001 Heverlee, Belgium

**Keywords:** Semiconductor lasers, Photonic crystals

## Abstract

The epitaxial growth of high-quality InGaAs/GaAs nano-ridges on silicon using aspect ratio trapping (ART) and nano-ridge engineering (NRE) has paved the way for the monolithic integration of laser sources on silicon. This breakthrough holds significant potential for integrated silicon photonics, enabling a wide range of applications and opening new research avenues. In this approach, the active material is grown not as a uniform layer but rather as parallel nano-ridge (NR) arrays. Leveraging this intrinsic feature of NRE, we propose a novel approach for realizing a surface-emitting laser and present the first experimental demonstration of this device. The device consists of an array of nano-ridges forming an in-plane cavity that can lase and couple light vertically. Based on an extensive design study, we demonstrate an optically pumped surface-emitting epitaxially grown nano-ridge laser (NRSEL) integrated on a 300 mm silicon wafer, which, to the best of our knowledge, is the first of its kind. We experimentally show lasing at the band edge of a photonic crystal by exploiting symmetry-protected bound states in the continuum (BICs). Additionally, we thoroughly characterize the far-field pattern. These findings lay the foundation for realizing high-density, integrated, and cost-effective electrically injected surface-emitting lasers on silicon.

## Abstract (web summary)

We demonstrate an optically pumped surface-emitting epitaxially grown nano-ridge laser (NRSEL) integrated on a 300 mm silicon wafer, which, to the best of our knowledge, is the first of its kind.

## Graphical abstract

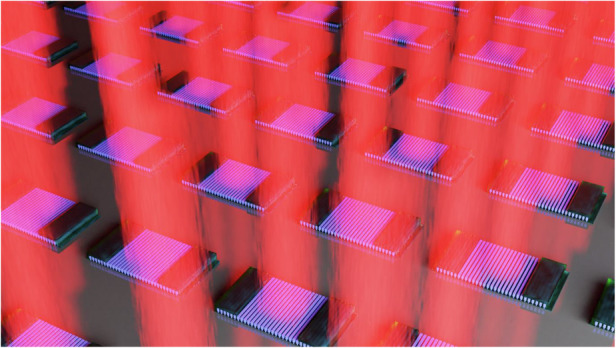

## Introduction

VCSELs (Vertical Cavity Emitting Lasers) are currently one of the most widely used optoelectronic devices. These compact lasers are used in datacom applications but also in consumer applications such as optical mice and smartphones, e.g for face recognition and ranging. VCSELs are fabricated from a complex vertical layer stack, consisting of a bottom DBR-mirror, a gain section, and a top DBR-mirror^1^. They can be fabricated and tested on wafer scale (typically on GaAs-substrates) and in general, are relatively cheap. However, they still suffer from a series of problems. VCSELS require complex epitaxial growth. The mirrors typically consist of 10–40 different layers, which must be grown with high accuracy. Only a few wavelengths can be addressed (most popular are 850 nm and 980 nm), as these are the ones where efficient DBR-mirrors can be relatively easily grown. Shifting to alternative wavelengths (e.g., 1300 nm, 1550 nm, MIR) is very challenging and not widely employed^2^. Another challenge is growing VCSELs with multiple wavelengths on the same wafer since the emission wavelength is determined by the growth and cannot easily be varied over the wafer in a controlled way. Monolithic integration with other photonic or electronic devices is difficult and typically not done.

**Fig. 1:**
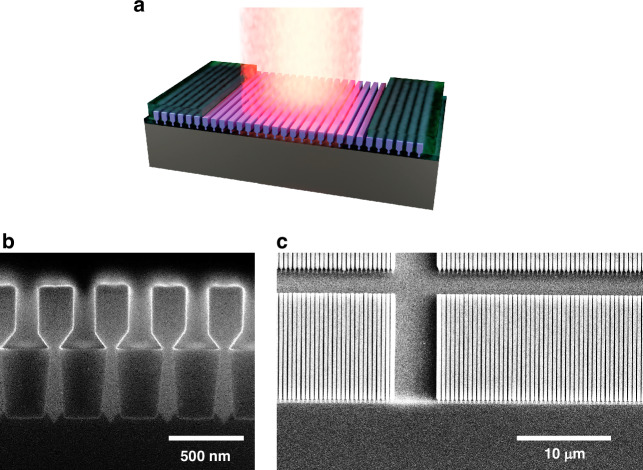
NRSEL on silicon. **a** Illustration of the proposed nano-ridge surface emitting laser (NRSEL). **b** Scanning electron microscope (SEM) image of the cross section of a cleaved array of nano-ridges. **c** Top-view SEM image of arrays of nano-ridges on a silicon wafer

Aspect ratio trapping (ART) and nano-ridge engineering (NRE) allow the growth of high-quality active materials with defect density^3^ below $6\times {10}^{4}c{m}^{-2}$ directly on silicon substrates without the need for a thick buffer layer^4,5^. Previously, we realized optically pumped DFB lasers and PIN-detectors based on this technology^6–9^. More recently, room-temperature electrically injected continuous-wave lasers were reported^3^, leveraging mode beating between the fundamental cavity mode and a higher order mode to mitigate losses linked to the electrical contacts. The active material is not grown as a uniform layer, but rather in the form of nano-ridges arranged in arrays (Fig. 1). These arrays can be considered as a high-index contrast photonic crystal supporting a series of optical modes^10–14^. In this paper we show that through careful design of the different device parameters, a slow light (band-edge) mode can be supported across an array of nano-ridges, forming a standing wave, providing enough feedback for lasing.

Nano-ridge based surface emitting lasers (NRSEL), as schematically presented in Fig. 1a, could present many advantages. Firstly, compared to VCSELS, which require several micrometer thick top and bottom DBR mirrors, the growth time is strongly reduced. Secondly, the emission wavelength can be tuned by changing the period of the array, the dimensions of the nano-ridges, or in post-processing by depositing additional materials (e.g. Silicon oxide, AlO_x_, or HfO_x_). Thirdly, the use of arrays with slightly varying periods makes it possible to create multi-wavelength laser arrays on a single die or wafer, while variations in material composition enable targeting different wavelength ranges. Moreover, the high index contrast of the photonic crystal promotes increased mode separation, ensuring dominance of a single mode within the gain bandwidth and thereby ensuring single mode operation without any competing modes. These advantages make the nano-ridge technology a promising platform for realising widely tunable, cost-effective, and CMOS-compatible surface-emitting lasers.

In this work, we first explain the basic operating principles of the device. Next, we study the different design parameters and their effects on the device performance. We also briefly discuss the nano-ridge epitaxy process and the laser cavity definition. Finally, we present the experimental results, obtained from optically pumped nano-ridge based surface emitting devices. We study the threshold behaviour as function of cavity size and discuss the spectral characteristics. Comparison with numerical calculations allows to match the observed peaks with the corresponding band edge modes in the band diagram. We also study the emission pattern and its divergence through back-focal plane imaging.

## Results

### Band-edge lasing

The array of nano-ridges (Fig. 1b, c) can be considered as a high-index contrast one-dimensional photonic crystal, inherently supporting multiple optical modes. The refractive index of the material and the index contrast play a crucial role in determining the band-gap^15,16^. Additionally, the height of the nano-ridge structure determines the number of supported modes. An increase in height results in the emergence of more bands, adding complexity to the band diagram. The periodicity and fill factor of the photonic crystal also influence the frequency of the modes. Notably, decreasing the period shifts the bands towards higher frequencies^16^.

To systematically track the optical modes of interest, a structured naming convention was employed. The modes appearing at the band-edge (band-edge modes) are denoted as *TE*_*xyz*_ and categorized based on the Bragg order (*x*), band number(*y*), and their position relative to the band gap (*z*), indicating whether they are predominantly confined in the dielectric region (lower band (*L*)) or the air region (higher band (*H*)). We focus on quasi TE-modes, having their electric field predominantly oriented along the nano-ridges, and hence within the plane of the quantum wells (QW). It is well-known that the compressively strained InGaAs QWs embedded within the GaAs nano-ridges provide the highest gain for this polarization^6,17^.

Figure 1a, b shows a schematic representation of the nano-ridge array and the band diagram calculated for an array of nano-ridges with period Λ = 380 nm, height H = 410 nm and width W = 197 nm. These dimensions were chosen to match the average dimensions of the waveguides in our fabricated sample, measured using SEM. Further information on the materials and simulation details can be found in the Methods section. The band diagram was calculated using a 2D finite difference time domain (FDTD) simulation with Bloch boundary conditions.

**Fig. 2:**
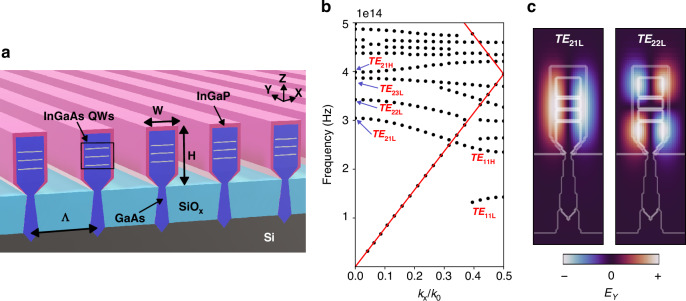
Nano-ridge 1D photonic crystal. **a** Schematic representation showing an array of nano-ridges with the most relevant dimensional parameters. **b** Dispersion diagram for an array of nano-ridges calculated using 2D-FDTD. The radiation continuum lies above the light line (red). **c** Calculated electric field for the first two band edge modes at the Γ point (k_*x*_/k_0_ = 0)

The band diagram demonstrates significant mode separation, which arises from the substantial contrast in refractive index between the air and dielectric sections of the photonic crystal^16^. Another result of the high index contrast is the flattening of the bands (reduced dispersion curvature) near the band edges^18,19^. At the band-edges, a slow-light Bloch mode (SBM) propagates inside the structure with group velocity *dω/dk* approaching zero due to the flat dispersion curvature. This phenomenon can be attributed to the superposition of reflected waves resulting from the index modulation - GaAs and air - at the symmetry points within the structure^11,16,20–22^.

We are interested in the modes at the high symmetry Γ point (*k*_*x*_ = 0) as these might couple to vertically emitting radiating modes. In Fig. 1c, the mode profiles for the first two band-edge modes at the Γ point, *TE*_21*L*_, and *TE*_22*L*_, are shown. Both modes are predominantly confined within the high-index dielectric region and are commonly referred to as dielectric modes. Typically, one expects that any mode whose dispersion line lies above the light line — that is, in the frequency–wavevector region where it can couple to free-space plane waves — will radiate away and thus have a finite lifetime. However, not all modes above the light line actually couple to free-space radiation. Some remain fully confined because they are symmetry-protected. Structural symmetries (such as mirror or rotational symmetry) can forbid coupling to radiative channels, effectively preventing these modes from leaking into the far field. As a result, they can exhibit very high or even theoretically infinite quality factors, despite lying in the radiative continuum. These modes are known as bound states in the continuum (BIC modes) and are characterized by their high Q-factor^23–25^. In a perfect and infinite photonic crystal of nano-ridges, *TE*_21*L*_ is considered a BIC mode, and cannot couple to radiating modes, despite being above the light line, due to the asymmetry of the mode’s electric field^10^, which has a node in the middle of the nano-ridge, as shown in Fig. 1c. Realistically, the photonic crystal is finite, leading to uncertainty in the in-plane wave vectors and hence symmetry breaking, allowing vertical emission^21^. For our nano-ridge height of 410 nm, two higher-order BIC modes^10^, *TE*_22*L*_ and *TE*_23*L*_ are also supported. They appear at higher energies and share the field antisymmetry that renders *TE*_21*L*_ a BIC. Since this symmetry protection only holds strictly at the Γ point, even a small shift of the in-plane wave vector degrades the confinement of all three modes. Consequently, these BIC modes exhibit a pronounced dependence on the in-plane wave vector: as one moves away from the Γ point, their Q-factor drops steeply, whereas the Q-factor of the first lossy (symmetric) mode shows only weak dependence on the in-plane wave vector^10,11,26,27^. A side-by-side comparison of the Q-factor dispersion and corresponding mode profiles for the first three BIC modes (*TE*_21*L*_, *TE*_22*L*_, and *TE*_23*L*_) and the first lossy mode (*TE*_21*H*_) is presented in Supplementary Information, Figure S1.

### Effect of the nano-ridge dimensions for infinite arrays

For optimising the design of the nano-ridges, we focused on the *TE*_21*L*_ mode, operating at the Γ point, as it provides the highest overlap with the quantum wells in the nano-ridge. Second, the nano-ridge dimensions and the period need to be chosen such that the resonance frequency of this mode matches the QW gain peak, around 1000 nm. Figure 2a shows how the resonance frequency of this mode is varying as function of period, fill factor and height. The fill factor is defined as the ratio of the nano-ridge width and the periodicity FF = W/Λ. The resonance frequency of the band-edge mode is extracted from the band diagram. The simulations were carried out using 2D-FDTD with Bloch boundary conditions. The period of the array obviously has the biggest impact on the resonance frequency. Also the nano-ridge width, and hence the fill factor have a strong influence. The effect of the nano-ridge height is less important. Height variations of 0.4–0.6 µm shift the resonance by only ~25–30 nm, whereas sweeping the width from 152 nm to 304 nm changes the resonance by roughly 375 nm, about an order of magnitude larger, making the nano-ridge width together with the periodicity the dominant parameters. The available mask (see section: Fabrication) provided only a fixed number of array designs, of which the one with a period of Λ= 380 nm provides the best match for our device design. Figure 2b shows that, with this period, the emission wavelength can be varied anywhere within the gain bandwidth of the QW’s, which ranges approximately from 980 nm to 1060 nm, by controlling the nano-ridge height and width.

**Fig. 3:**
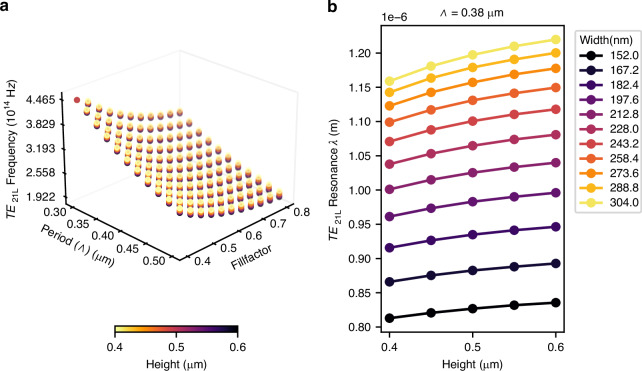
Geometry dependent resonance frequency shift. **a** Sweep of the resonance frequency of the *TE*_21*L*_ mode, as function of nano-ridge array period (Λ) and fill factor, for varying nano-ridge heights (*H*). **b** Resonance wavelength (*λ*) for the chosen design period Λ = 0.38 µm, as function of height (*H*), for different widths (*W*)

### Design of Nano-Ridge Surface Emitting Laser (NRSEL) with finite dimensions

In the previous section we considered a nano-ridge array with infinite dimensions. Although we did obtain laser operation of quasi-infinite arrays (see Fig. 8 and supplementary information figures S2, S3), in practice we desire to keep the dimensions more restricted.

Reducing the dimensions of the NRSEL array adversely impacts the device performance however. A reduction in the number of periods results in decreased confinement and in-plane light leakage. This is clearly visible from Fig. 3b, which shows a 2D FDTD simulation of the TE_21*L*_ mode for an array of 40 nano-ridges with Λ = 380 nm, H = 410 nm and W = 197 nm. Figure 3c shows how the Q-factor of this mode increases with an increasing number of nano-ridge periods, and saturates for more than 90 periods, when the in-plane losses become negligible. As the modal gain threshold is inversely proportional to the Q-factor G_th_ ∝ 1/Q, reaching a low threshold requires a relatively large device. This trade-off can be overcome by introducing suitable mirrors at both sides of the cavity. A possible approach consists of forming a heterostructure from two photonic crystals with shifted bandgap, as shown schematically in Fig. 3a. If one ensures that the frequency of the targeted band-edge mode of the central photonic crystal falls within the bandgap of the surrounding photonic crystal, the latter acts as a mirror and reduces in-plane losses. This approach allows to localize the mode while keeping the Q-factor sufficiently high, thereby allowing for low threshold lasing^28–30^. The standard approach to shift the bandgap of a photonic crystal is to change its period. However, due to a limitation in the available mask used for patterning the nano-ridge we choose a different approach here. Specifically, depositing a material with refractive index higher than air, such as SiO_2_, between the nano-ridges shifts the bandgap frequency downwards, as illustrated in Fig. 3a. Simulations show that embedding an array of nano-ridges within a 1.5 µm thick photoresist layer with refractive index 1.6, shifts the resonance wavelength of the *TE*_21*L*_ mode from 984 nm to 1012 nm.

**Fig. 4:**
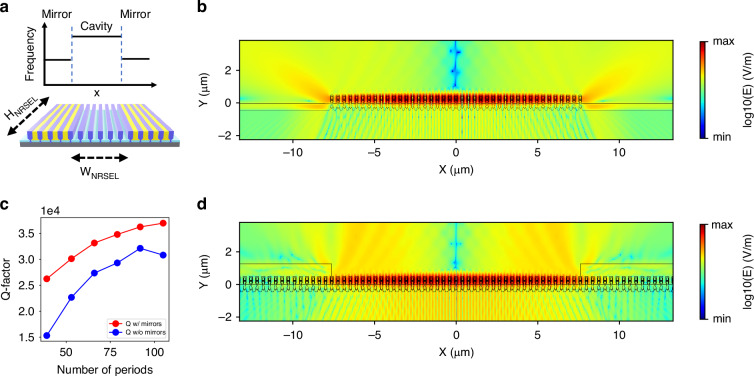
Bloch mode confinement with side mirrors. **a** Side mirrors defined by locally shifting the slow Bloch mode to a longer wavelength. This forms a barrier around the middle cavity, enhancing the electric field in the cavity, and reducing the lateral losses. **b**, **d** A 2D FDTD simulation of a finite nano-ridge crystal without and with mirrors, showing a cross-section of the TE_21L_ mode. The side mirror is formed by adding photoresist of index 1.6 between and on top of the nano-ridges. **c** Calculated Q-factor for the TE_21L_ mode without and with side mirrors, as function of the number of periods

Figure 3d shows the simulated mode profile for a cavity formed by a central array of 40 uncovered nano-ridges and mirrors of 15 nano-ridges embedded within SiO_2_. The reduced lateral leakage, increased confinement and reflection at the boundaries is clearly visible. Adding the mirror increases the Q-factor from 15,000 to 26,000 for the 40 periods device. The vertical emission increases to 20.7% compared to around 14.3% without the side oxide mirrors. Adding a bottom mirror could further increase the vertically coupled power. Note that the upwards radiating field shows a node in the centre, originating from the asymmetry of the *TE*_21*L*_ mode mentioned above. In future designs this could be avoided using earlier reported strategies^31–33^ but this is not further considered here.

### Fabrication

#### Nano-Ridge epitaxy

The InGaAs/GaAs nano-ridges were epitaxially grown on 300 mm patterned silicon wafers using NRE. This involves a combination of selective area growth in narrow trenches to confine relaxation defects arising from the large lattice mismatch via aspect ratio trapping (ART), and nano-ridge shape engineering during the growth out of the trench, to control the final shape of the nano-ridges. The process starts with the formation of Si fins embedded in SiO_2_, which are realized via a shallow trench isolation (STI) process on (001) Si, a common approach in CMOS fabrication. These Si fins are then wet-etched with tetramethylammonium hydroxide to expose two {111} Si facets required for subsequent III-V growth without antiphase disorder. Then MOVPE is used to grow the InGaAs/GaAs nano-ridges. The application of very narrow trenches prevents threading dislocations from protruding into the active InGaAs QW layers. Once the material extends above the SiO_2_ mask, careful control of the growth parameters allows to obtain the box-like shape visible in Fig. 4b with dimensions as obtained from simulations. Three In_0.23_Ga_0.77_As QWs are embedded in the GaAs nano-ridges for providing optical gain. We estimated an indium content of 23 ± 1% and a quantum well thickness of 11 ± 1 nm. To improve the carrier confinement and to reduce surface recombination, the InGaAs/GaAs nano-ridges were capped with a lattice matched InGaP layer. The nano-ridges were grown in blocks of 5 mm × 5 mm within which the trench size and period is held constant, ensuring good uniformity, as illustrated in Fig. 4b, c. The mask design included multiple trench periods, but from the simulations shown in Fig. 2 we found that only the 380 nm period matches the gain bandwidth of the nano-ridges. Using Focused Ion Beam (FIB) milling, a cross-section of the nano-ridge array was made, and the dimensions of the nano-ridge were measured to be H = 410 nm and W = 197 nm. These dimensions were used in the band diagram and Q-factor simulations presented earlier. To define localized NRSEL devices, further processing is required, as discussed in the next section.

#### NRSEL definition

**Fig. 5:**
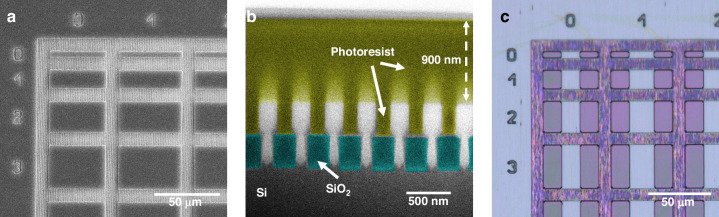
NRSEL fabrication. **a** SEM image showing definition of the NRSEL array after successful etching of the nano-ridge material using ICP. **b** False coloured cross-section SEM image after defining the side mirrors using photoresist, showing successful infilling and coverage with a height of 900 nm on top of the nano-ridges. **c** Optical image after NRSEL definition and development of the side mirrors

The goal is to define localized nano-ridge surface-emitting lasers (NRSELs) with controlled dimensions. To simplify the process and as a proof-of-principle, we used photoresist instead of SiO_2_ to form the mirrors. The photoresist used (AZ 5214) has a refractive index close to *n* = 1.6 and can easily be patterned using standard optical lithography. First arrays of NRSEL devices with different widths and heights were defined in a AZ5214 photoresist layer using optical lithography. Subsequently the nano-ridge material outside the patterned NRSELs was etched away using a SiCl_4_/N_2_-based Inductively Coupled Plasma Etching (ICP RIE) process, as shown in Fig. 5a. In a following step, the mirrors were defined by spin coating photoresist (AZ 5214), which was then patterned through optical lithography. Figure 5b shows a cross-section of the mirror section, illustrating that the photoresist provides good filling between the nano-ridges. Figure 5c is a microscope picture of the NRSEL array, showing the final NRSEL devices. In the design, the width of the side mirrors was kept constant at 15 µm while the cavity width W_*NRSEL*_ was varied from 15 to 40 µm. The height of the cavity H_*NRSEL*_ was varied from 5 to 45 µm.

### Measurements

**Fig. 6:**
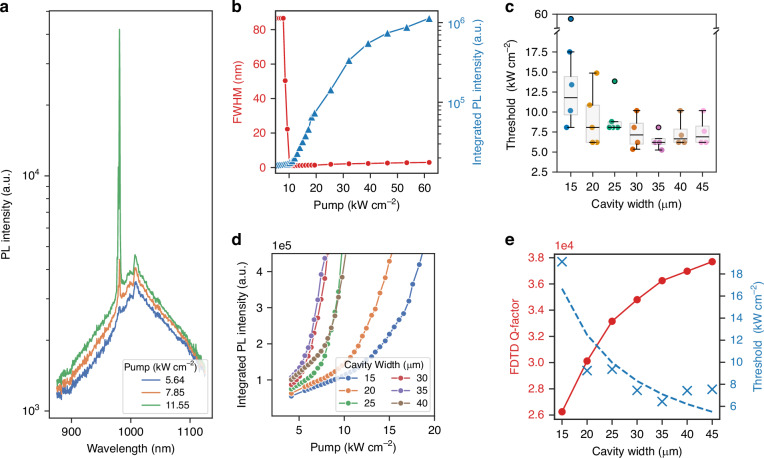
Room-temperature optical characterization. **a** PL spectra of W_*NRSEL*_ = 20 µm NRSEL for different pump intensities. **b** Integrated PL and the line width for a NRSEL of W_*NRSEL*_ = 20 µm as function of pump power. The line width reaches a minimum of 0.9 nm at threshold. **c** Measured lasing threshold versus cavity width for 31 devices within the same sample. Individual scatter points represent the threshold of each device, while the overlaid box plots summarize the statistical distribution of thresholds for each cavity width. The solid black line in each box represents the median value of the data. **d** Zoom in on the integrated PL for a group of six NRSELs with different widths shows the reduction of the threshold with the increase in cavity width. **e** The threshold data is fitted using a reciprocal function, a/(W_*NRSEL*_)+b. Scatter points (blue) represent the mean threshold values, and the calculated Q-factor (red) is plotted for comparison

The fabricated samples were measured using micro-photoluminescence (µPL) spectroscopy at room temperature with a 532 nm nanosecond pulsed Nd:YAG laser as the pump source. The laser was focused into a 300 µm diameter spot using a microscope objective with NA = 0.65. The emission from the devices was collected through the same microscope objective with the help of a dichroic mirror, and detected with a spectrometer (KYMERA-328I-D2-SIL, Oxford instruments, Andor) that has two sensors attached: a water-cooled InGaAs detector (iDus, DU490A-1.7 Model, Oxford Instruments, Andor) and a visible range CCD sensor (iDus 401, DU401A). First, we characterized a NRSEL device of width *W*_*NRSEL*_ = 20 µm and height *H*_*NRSEL*_ = 15 µm in detail, measuring its PL spectra under different pumping conditions, from which we determined its threshold pump density and its linewidth. The results are shown in Fig. 6a, b. Then, 31 devices with varying cavity widths were characterized to explore the effect of cavity width on the lasing threshold and to compare the results with the simulated Q-factors presented earlier (Fig. 6c–e). Finally, a NRSEL device of width *W*_*NRSEL*_ = 35 µm and an unpatterned region, which can be considered as a quasi-infinite array of nano-ridges (Fig. 8c), were further investigated using back focal plane (BFP) imaging, a technique also known as fourier plane imaging (Fig. 8d, e).

### Laser characterization

Figure 6a shows the measured spectra under various pumping conditions for an NRSEL with W_*NRSEL*_ = 20 µm. At low pump intensities, a broad spontaneous emission spectrum is observed. When increasing the pump power, a lasing peak emerges (*λ* = 984 nm). Figure 6b, shows the measured linewidth and the integrated PL intensity around the lasing peak as function of the pump power. The lasing threshold is calculated by taking the intersection point between the fit of the linear region of the integrated PL signal and the lower axis (pump power). For this device, the threshold pump density is 10.8 kW cm^−^^2^. The narrowest linewidth is obtained just above threshold and is measured to be 0.9 nm. We believe the linewidth to be limited by the pulsed optical pumping.

Figure 8a shows a microscope image of the NRSEL with W_*NRSEL*_ = 20 µm under optical pumping above the lasing threshold. The pump light is filtered out using a dichroic mirror. Note that the pump spot is much larger than the cavity size. The picture shows clear confinement of the optical mode within the central cavity as defined by the photoresist mirrors, indicating successful fabrication. The emission is highly polarized, with the electric field aligned within 20° of the nano-ridge axis. This confirms the mode is traveling perpendicular to the nano-ridges and does not simply originate from reflections at the top and bottom facets of the nano-ridge. More details are discussed in the supplementary information (S5). For this device, also localised light emission from the mirror region can be seen. This emission appears at higher pump powers and is not expected, as the mirror regions have a lower index contrast and a smaller width compared to the central cavity, which should lead to higher losses and a lower Q-factor. This effect is not observed for all cavities: e.g., for the device with W_*NRSEL*_ = 25 µm, shown in Fig. 8b, only emission from the central cavity is observed. Therefore, we believe it is associated with variations in the nano-ridge’s dimensions and imperfections that can lead to the localization of light emission in some parts of the array. The nature of this emission and the associated SBM will be discussed in more detail below.

To evaluate the effect of the cavity width on the devices’ lasing threshold, we compared a group of devices with W_*NRSEL*_ varying from 15 to 45 µm. Figure 6d shows the associated light-in vs. light-out curves for a group of six devices and Fig. 6c shows how the threshold pump density varies with the cavity width for all the measured devices. In total 31 devices were measured. The experimental data reveals an inverse relationship: as the cavity width increases from 15 to 35 µm, there is a sharp reduction in the threshold (up to a 63% drop). Beyond 35 µm, however, the trend reverses slightly: the lasing thresholds increase by approximately 14%. This inflection appears to coincide with a regime where the simulated Q-factor—although still rising from about 36,300 to 37,700 as shown in Fig. 6e—starts to saturate. In this regime, further widening of the cavity yields only marginal improvements in the Q-factor (roughly an additional 4%). The saturation of the threshold might indicate the influence of random disorder effects, as longer cavity photonic crystal lasers tend to suffer higher losses (often referred to as slow-light induced losses^34^) when operating near the band-edge. Notably, the minimum lasing threshold was found for devices with a 35 µm cavity, which also exhibited the smallest spread in threshold values—suggesting that the optimal cavity dimension minimizes the impact of fabrication-induced disorder. The spread in threshold for devices of the same cavity size can be attributed to disorder and slight changes in dimensions. Overall, while increasing the cavity width leads to lower lasing thresholds due to improved Q-factors, the practical realization of these benefits is highly sensitive to fabrication tolerances and disorder.

To study dimensional non-uniformity, we scanned the pump spot across a 700 µm region of an unpatterned sample (Supplementary Information S3). The scan revealed a −35 nm wavelength shift from the edge to the center of the sample. Separate SEM measurements show the nano-ridge width increasing from 216 ± 2 nm at the edge to 226 ± 5 nm at the center. Using the FDTD-calculated sensitivity of 2.5 nm of spectral shift per nanometre of width, this 10 nm change in dimensions is expected to result in a 25 nm wavelength shift. Given the measurement uncertainty, the experimental and simulated results are in good agreement. These variations not only shift the band-edge wavelength but also affect the cavity Q factor, with simulations indicating an approximate 34% change. Figure 8c shows a real-space image of the unpatterned region, showing the laser emission from a quasi-infinite array of nano-ridges.

**Fig. 7:**
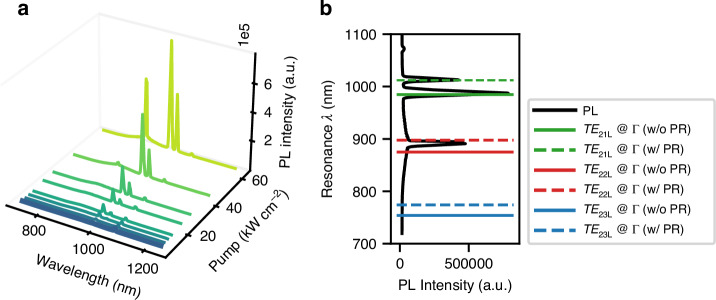
Mode identification for an NRSEL pixel with W_NRSEL_ = 20 µm. **a** PL spectra for increasing pump power. For the highest pump power, three distinct peaks are visible. **b** Comparison between the experimental PL spectrum taken at 62.4 kW cm^−^^2^ pump power density and the calculated wavelengths of the *TE*_21*L*_, *TE*_22*L*_ and *TE*_23*L*_ modes, at the Γ point in the band diagrams for a nano-ridge array without photoresist and with photoresist

**Fig. 8:**
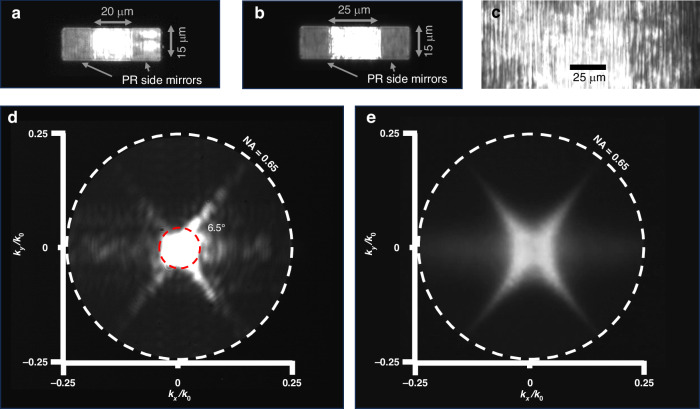
Real space and fourier plane imaging. **a**, **b** Real-space images of devices with cavity sizes of 20 µm and 25 µm under optical pumping above the lasing threshold. In the 20 µm device, light is mostly confined to the cavity, although some emission extends into the photoresist mirror region on the right. In contrast, for the 25 µm device the emission is entirely localized within the cavity, with a clear reflection observed at the interface between the cavity and the photoresist side mirrors. **c** Real-space image of a quasi-infinite array under optical pumping above the lasing threshold, showing the emission from a large area of nano-ridges. **d** Back focal plane image for the 25 µm device above the lasing threshold, showing an angular divergence of approximately 6.5°. **e** Back focal plane image of the quasi-infinite array above the lasing threshold

To unambiguously match the peaks visible in the laser spectrum with the modes found in the simulated band-diagram, the NRSEL with W_*NRSEL*_ = 20 µm was characterised again, now using the visible range CCD sensor to be able to measure wavelengths shorter than 900 nm. The result is shown in Fig. 7. As discussed above, this device shows emission from both the mirror region and the cavity region (see Fig. 8a) and the spectra shown here include light from both regions. With increasing pump power, first a peak at 985 nm starts to appear, then a second one at 1010 nm, and lastly a third one around 890 nm. In Fig. 7b, c, these experimental results are compared with FDTD simulations of the band diagram for nano-ridge arrays with and without photoresist. Figure 7b shows the measured spectrum at high pump power (62.4 kW cm^−^^2^) and Fig. 7c shows the wavelength of the *TE*_21*L*_, *TE*_22*L*_ and *TE*_23*L*_ modes, at the Γ point. The first peak in the lasing spectrum, at 985 nm, matches the *TE*_21*L*_ mode of the array without PR. The calculated Q-factor of this mode is 26,053 (for a cavity size of 40 nano-ridges with mirrors). The second peak, at 1010 nm, matches the *TE*_21*L*_ mode for a nano-ridge array with photoresist. Using a diaphragm, we could indeed associate this mode in the spectrum with emission coming from the photoresist region. Given this mode’s lower Q-factor (9200) it shows up at higher pump powers. The third peak (890 nm) matches the higher order *TE*_22*L*_ mode. This mode has a relatively low Q-factor of 4056. Additionally, its wavelength lies outside the quantum well gain region. Therefore, we suspect that the gain experienced by this mode originates from the GaAs nano-ridge itself. Note that the small deviation between the measured and simulated wavelengths can be explained by small deviations in the nano-ridge dimensions or values of the refractive index.

The devices were studied further using back-focal plane (BFP) imaging. The BFP of an objective lens contains information about the angular spectrum of the light emitted from the sample plane. By imaging the BFP, the angular distribution of light emitted from the photonic crystal can be mapped to momentum space using the formula: *k*_*//*_ = *k*_0_*sin*(*θ*). Hence, the in-plane k-vector (*k*_*//*_) can be evaluated knowing the emission wavelength (*k*_0_ = 2*π/λ*) and the emission angle (*θ*), theta is the angle between the normal to the surface and the propagation direction of the emitted light. This mapping is crucial for understanding the dispersion relations in photonic crystals^35,36^. Figure 8d, e, show the back-focal plane image of the NRSEL with W_*NRSEL*_ = 25 µm and the quasi-infinite array shown in Fig. 8b, c respectively, above their respective lasing threshold. Both devices show an angle of divergence lower than 10^◦^, reflecting relatively tight beam confinement.

The unique emission patterns shown in Fig. 8d, e represent iso-frequency contours of the three-dimensional dispersion surface^37^ (see Supplementary Information S3). Above the lasing threshold, the intense monochromatic emission at the Γ point couples to modes at the same frequency within the band diagram, resulting in the distinctive star-shaped emission pattern observed. For the quasi-infinite array, the emission pattern clearly shows the characteristic star-shaped pattern also found from the 3D band structure calculations (see figure S3). For the finite size cavity, this pattern is still visible but now additional k-vector components are added, due to the scattering induced by the mirrors, as can be seen in Fig. 3d.

## Discussion

We introduced a novel concept for realizing a surface emitting nano-ridge laser (NRSEL) epitaxially grown on a trench patterned 300 mm silicon wafer. These devices experimentally showed stimulated emission with thresholds as low as 5 kW cm^−^^2^ and single mode operation. We numerically demonstrate how the emission wavelength can be tuned by adjusting the nano-ridge width, with FDTD simulations predicting a sensitivity of 2.5 nm nm^−^¹. Photoluminescence and SEM measurements show a shift of 35 nm in the peak wavelength and a change of 10 ± 5 nm in the nano-ridge width going from the edge to the center of the sample and hence a sensitivity of 3.5 nm nm^−^¹, confirming the numerical trend. Also, the far-field pattern was characterised in depth. We believe these NRSELs offer a huge potential for a wide range of applications, possibly overcoming some of the limitations suffered today by VCSELs in terms of accessible wavelength range and scalability. Given that they are directly integrated on a standard silicon substrate, integration with other photonic or even electronic devices can be envisaged. By changing the material composition, a wide spectral range can be addressed. E.g., we have shown before that modifying the indium content to *In*_0.45_*Ga*_0.55_*As* enables shifting the emission to 1.3 µm^9^. Integrating alternative material systems, such as GaSb^38^, might even allow for the design of lasers emitting at wavelengths beyond 1.5 µm. Recently, we have also demonstrated electrically injected nano-ridge lasers^3^, by introducing a p-i-n junction in the nano-ridge. In this device, the current was injected through tungsten plugs on top of the nano-ridge and on the n-doped silicon substrate, fabricated using a standard damascene process. In principle, such an approach should also be viable for the NRSEL proposed here. However, care should be taken to not disturb the mode profile and to not block the emitted light with the top electrodes. For small devices, preliminary simulations show the electrodes could be placed at the side, while still ensuring a uniform current injection. For larger devices, an approach similar to that of the electrically injected single nano-ridge laser^3^ might be taken, whereby the plugs are located at the nodes of the optical field, to avoid excessive optical losses. In summary, we have proposed and validated a new and highly versatile concept for realizing vertically emitting lasers, with potential applications in telecommunications, LIDARs, environmental sensing, and spectroscopy.

## Materials and Methods

### Numerical simulations

The band diagrams and mode profiles were calculated using the 2D finite-difference time-domain method (Lumerical FDTD). To simulate an infinite array of nano-ridges, a single nano-ridge was simulated with Bloch boundary conditions applied in the direction of the periodicity and excited using randomly oriented dipole sources. For the Q-factor calculations, finite arrays of nano-ridges with PML boundary conditions at the sides were excited using a dipole source. The nano-ridge dimensions used in the simulations are the average dimensions as measured using SEM for the fabricated sample: period Λ = 380 nm, height H = 410 ± 3.5 nm and width W = 197 ± 10 nm. For GaAs and Silicon, default material parameters from Lumerical’s material library were used (Handbook of Optical Constants of Solids I - III by E. Palik). For the InGaP layer, a refractive index of 3.2193 was used, with a thickness of 30 nm on the sidewalls and 60 nm on the top of the nano-ridge. These thicknesses are included in the overall nano-ridge width and height. InGaAs is assigned a refractive index of 3.6339. SiO₂ is assigned a refractive index of 1.4498.

### Epitaxy

The III-V material was deposited in a commercial 300 mm single wafer MOVPE reactor using standard metal organic precursors such as trimethylgallium (TMGa), triethylgallium (TEGa), trimethylindium (TMIn), tertiarybutyl arsine (TBAs) and tertiarybutyl phosphine (TBP). The GaAs nucleation was done at 360 °C applying TEGa and TBAs, then the growth temperature was raised to 590 °C for the GaAs trench filling using TMGa and TBAs. Subsequently, the temperature was set to 560 °C for the growth of the compressively strain InGaAs/GaAs multi-quantum well stack with the TMIn/TMGa+TMIn ratio adjusted to achieve an In concentration of approximately 20–22%. Finally, the temperature was increased to 600 °C for the InGaP capping layer, with an In composition of 51% to ensure it was lattice-matched to GaAs. The TBAs/III and TBP/ III ratios were varied between 10 and 60 as a function of the material and growth temperature.

### Device Patterning

The sample was prepared by spin-coating AZ 5214 photoresist at an acceleration of 1000 RPM/s and a speed of 4000 RPM for 40 seconds, followed by a soft bake at 100 °C for 3 minutes. After baking, the photoresist was exposed to UV light for 30 seconds to define the desired pattern. The exposed regions were developed in AZ 400 K developer, diluted 1:3 with water, for 20 s to reveal the features. At this stage, the process could proceed with ICP etching for etching away the nano-ridges or stop here for the fabrication of side mirrors. Finally, after ICP etching the photoresist was removed by immersing the sample in AZ 100 resist remover at 80 °C for 10 min.

### Optical characterization

The fabricated samples were characterized using micro-photoluminescence spectroscopy at room temperature. A 532-nm nanosecond pulsed Nd-YAG laser, with a pulse width of 7 ns and a repetition rate of 1000 Hz, was used as the optical pump. The laser focused into a spot with a diameter of 300 µm using a microscope objective with a numerical aperture (*NA*) of 0.65. The emission from the devices was collected through the same microscope objective with the help of a dichroic mirror and detected with a spectrometer (KYMERA-328I-D2-SIL, Oxford instruments, Andor) that has two sensors attached at different output paths: a water-cooled InGaAs detector (iDus, DU490A-1.7 Model, Oxford Instruments, Andor) and a visible range CCD sensor (iDus 401, DU401A). For the Fourier imaging an additional focusing lens is used to image the back-focal plane of the microscope objective on a separate CMOS camera.

## Supplementary information

Supplementary Information

## Data Availability

The datasets generated and analyzed during the current study are available from the corresponding authors upon reasonable request.

## References

[1] Wang, S. C. et al. Optically pumped GaN-based vertical cavity surface emitting lasers: technology and characteristics. *Jpn. J. Appl. Phys.* **46**, 5397–5407 (2007).

[2] Belkin, M. E. et al. Long wavelength VCSELs and VCSEL-based processing of microwave signals. in Optoelectronics - Materials and Devices (eds Pyshkin, S. L. & Ballato, J.) (Rijeka: IntechOpen, 2015).

[3] De Koninck, Y. et al. GaAs nano-ridge laser diodes fully fabricated in a 300-mm CMOS pilot line. *Nature* **637**, 63–69 (2025). 39743604 10.1038/s41586-024-08364-2

[4] Kunert, B. et al. III/V nano ridge structures for optical applications on patterned 300 mm silicon substrate. *Appl. Phys. Lett.* **109**, 091101 (2016).

[5] Kunert, B. et al. (Invited) integration of III/V hetero-structures by selective area growth on Si for nano- and optoelectronics. *ECS Trans.* **75**, 409–419 (2016).

[6] Shi, Y. T. et al. Optical pumped InGaAs/GaAs nano-ridge laser epitaxially grown on a standard 300-mm Si wafer. *Optica* **4**, 1468–1473 (2017).

[7] Shi, Y. T. et al. Loss-coupled DFB nano-ridge laser monolithically grown on a standard 300-mm Si wafer. *Opt. Express* **29**, 14649–14657 (2021). 33985182 10.1364/OE.422245

[8] Ozdemir, C. I. et al. Low dark current and high responsivity 1020 nm InGaAs/GaAs nano-ridge waveguide photodetector monolithically integrated on a 300-mm Si wafer. *J. Lightwave Technol.* **39**, 5263–5269 (2021).

[9] Colucci, D. et al. Unique design approach to realize an O-band laser monolithically integrated on 300 mm Si substrate by nano-ridge engineering. *Opt. Express* **30**, 13510–13521 (2022). 35472961 10.1364/OE.454795

[10] Chen, M. H. et al. GaN ultraviolet laser based on bound states in the continuum (BIC). *Adv. Optical Mater.* **11**, 2201906 (2023).

[11] Le, N. D. et al. Super bound states in the continuum on a photonic flatband: concept, experimental realization, and optical trapping demonstration. *Phys. Rev. Lett.* **132**, 173802 (2024). 38728718 10.1103/PhysRevLett.132.173802

[12] Bogaerts, W. et al. Out-of-plane scattering in 1-D photonic crystal slabs. *Optical Quantum Electron.* **34**, 195–203 (2002).

[13] Ovcharenko, A. I. et al. Bound states in the continuum in symmetric and asymmetric photonic crystal slabs. *Phys. Rev. B* **101**, 155303 (2020).

[14] Johnson, S. G. et al. Guided modes in photonic crystal slabs. *Phys. Rev. B* **60**, 5751–5758 (1999).

[15] Cavalcanti, S. B. et al. Band structure and band-gap control in photonic superlattices. *Phys. Rev. B* **74**, 153102 (2006).

[16] Joannopoulos, J. D. et al. Photonic Crystals: Molding the Flow of Light. 2nd edn. (Princeton: Princeton University Press, 2008).

[17] Chuang, S. L. Physics of Photonic Devices. 2nd edn. (New York: John Wiley & Sons, 2009).

[18] Nojima, S. Optical-gain enhancement in two-dimensional active photonic crystals. *J. Appl. Phys.* **90**, 545–551 (2001).

[19] Droulias, S. et al. Lasing threshold control in two-dimensional photonic crystals with gain. *Opt. Express* **22**, 19242–19251 (2014). 25321009 10.1364/OE.22.019242

[20] Sakai, K. et al. Lasing band-edge identification for a surface-emitting photonic crystal laser. *IEEE J. Sel. Areas Commun.* **23**, 1335–1340 (2005).

[21] Kwon, S. H. & Lee, Y. H. High index-contrast 2D photonic band-edge laser. *IEICE Trans. Electron.* **E87-C**, 308–315 (2004).

[22] Viktorovitch, P. et al. Photonic crystals: basic concepts and devices. *Comptes Rendus Phys.* **8**, 253–266 (2007).

[23] Hsu, C. W. et al. Bound states in the continuum. *Nat. Rev. Mater.* **1**, 16048 (2016).

[24] Zhen, B. et al. Topological nature of optical bound states in the continuum. *Phys. Rev. Lett.* **113**, 257401 (2014). 25554906 10.1103/PhysRevLett.113.257401

[25] Hwang, M. S. et al. Ultralow-threshold laser using super-bound states in the continuum. *Nat. Commun.* **12**, 4135 (2021). 34226557 10.1038/s41467-021-24502-0 PMC8257597

[26] Kodigala, A. et al. Lasing action from photonic bound states in continuum. *Nature* **541**, 196–199 (2017). 28079064 10.1038/nature20799

[27] Le, N. D. et al. Extended Bound states in the Continuum for ultraheavy photons in photonic lattice. Print at 10.48550/arXiv.1905.00215 (2019).

[28] Inoue, T. et al. Design of photonic-crystal surface-emitting lasers with enhanced in-plane optical feedback for high-speed operation. *Opt. Express* **28**, 5050–5057 (2020). 32121733 10.1364/OE.385277

[29] Ferrier, L. et al. Slow Bloch mode confinement in 2D photonic crystals for surface operating devices. *Opt. Express* **16**, 3136–3145 (2008). 18542400 10.1364/oe.16.003136

[30] Viktorovitch, P. et al. Double photonic crystal vertical-cavity surface-emitting lasers. Proceedings of SPIE 8633, High Contrast Metastructures II. San Francisco, California, United States: SPIE, 863302 (2013).

[31] Li, S. et al. Analysis of surface-emitting second-order distributed feedback lasers with central grating phaseshift. *IEEE J. Sel. Top. Quantum Electron.* **9**, 1153–1165 (2003).

[32] Jin, Y. et al. High power surface emitting terahertz laser with hybrid second- and fourth-order Bragg gratings. *Nat. Commun.* **9**, 1407 (2018). 29643341 10.1038/s41467-018-03697-9 PMC5895695

[33] Biasco, S. et al. Highly efficient surface-emitting semiconductor lasers exploiting quasi-crystalline distributed feedback photonic patterns. *Light Sci. Appl.* **9**, 54 (2020). 32284856 10.1038/s41377-020-0294-z PMC7142150

[34] Xue, W. Q. et al. Threshold characteristics of slow-light photonic crystal lasers. *Phys. Rev. Lett.* **116**, 063901 (2016). 26918991 10.1103/PhysRevLett.116.063901

[35] Le Thomas, N. et al. Exploring light propagating in photonic crystals with Fourier optics. *J. Optical Soc. Am. B* **24**, 2964–2971 (2007).

[36] Le Thomas, N. et al. Fourier space imaging of light localization at a photonic band-edge located below the light cone. *Phys. Rev. B* **79**, 033305 (2009).

[37] Regan, E. C. et al. Direct imaging of isofrequency contours in photonic structures. *Sci. Adv.* **2**, e1601591 (2016). 28138536 10.1126/sciadv.1601591 PMC5262448

[38] Baryshnikova, M. et al. Nano-ridge engineering of GaSb for the integration of InAs/GaSb heterostructures on 300 mm (001) Si. *Crystals* **10**, 330 (2020).

