# Supplementary material for: One-dimensional photonic crystal nano-ridge surface emitting lasers epitaxially grown on a standard 300 mm silicon wafer
Source: Light Sci Appl. 2026 Feb 24;15:120. doi: 10.1038/s41377-025-02061-z (PMC12932861; doi:10.1038/s41377-025-02061-z)
Supplement: Supplementary file 1 — Supplementary Information [file 41377_2025_2061_MOESM1_ESM.docx]

**Supplementary Information for:** **One-Dimensional Photonic Crystal Nano-Ridge Surface Emitting Lasers Epitaxially Grown on a Standard 300mm Silicon Wafer**

**Eslam M.B. Fahmy**^1,*^**, Zhongtao Ouyang**^1^**, Davide Colucci**^2^**, Nicolas Le Thomas**^1^**, Joris Van Campenhout**^2^**, Bernardette Kunert**^2^**, and Dries Van Thourhout**^1^

**BIC modes vs leaky mode Q-factor**


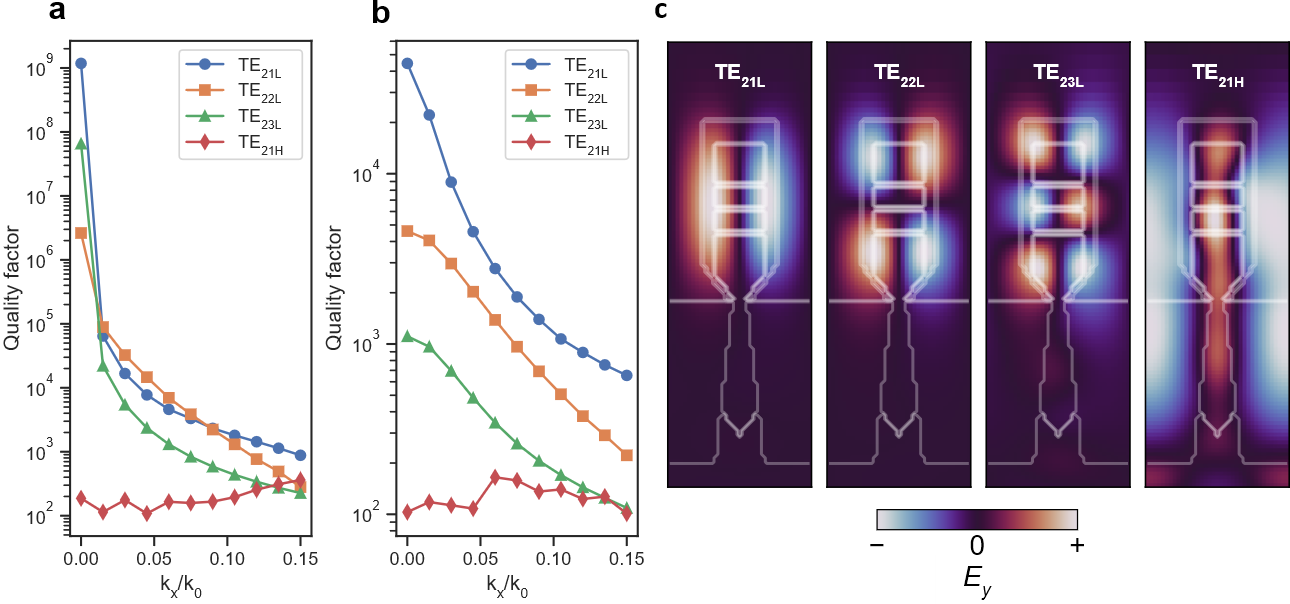


S1. a) Quality factor for an array of nano-ridges without a silicon substrate using bloch boundary conditions calculated for different k values. For the asymmetric BIC modes *TE*_21_*_L_*, *TE*_22_*_L_*, and *TE*_23_*_L_* the quality factor drops significantly off-gamma ($k_{x}$=0). The leaky symmetric mode *TE*_21_*_H_* has a low quality factor and a weak dependence of the quality factor on the k value. b) Quality factor for an array of nano-ridges with a silicon substrate using bloch boundary conditions calculated for different k values. For the asymmetric BIC modes *TE*_21_*_L_*, *TE*_22_*_L_*, and *TE*_23_*_L_* the quality factor drops significantly off-gamma ($k_{x}$=0). The substrate limits the maximum achievable quality factor as it allows leakage.

**Lasing from Quasi-Infinite Arrays**

A quasi-infinite nano-ridge array of period Λ = 380 nm was optically pumped at different pump powers. The pump laser was focused into a pump spot with a diameter of 300 µm. As shown in figure S1, upon reaching a pump power density of 10 kW cm^-2^, a sharp peak appears at 986 nm, corresponding to the BIC *TE*_21_*_L_* mode of interest.

**
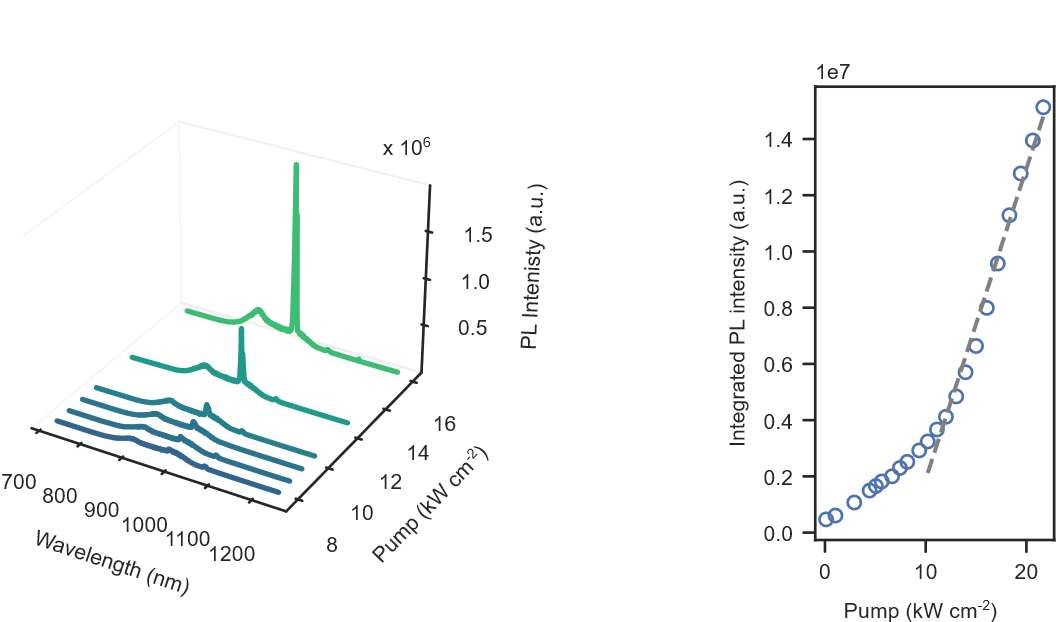


S2.** a) Lasing spectrum of a quasi-infinite array of period Λ = 380 nm, pumped with a spot size of 300 µm diameter at different pump power densities. Above the lasing threshold a narrow lasing peak appears at 984 nm. b) Integrated PL intensity showing a lasing threshold around 10 KW cm^-2^.

To study the uniformity of the sample, measurements were taken at regular positions across the sample surface. In this case we used a square pump spot size of 90 µm x 90 µm. Fig. S2a and b show normalized lasing PL spectra and the corresponding peak wavelength, taken at different positions, starting near the edge of the array and moving inwards. This measurement reveals a band-edge wavelength shift of approximately 35 nm over a 700 µm span, indicating variations in waveguide dimensions on the order of ±10 nm.

**
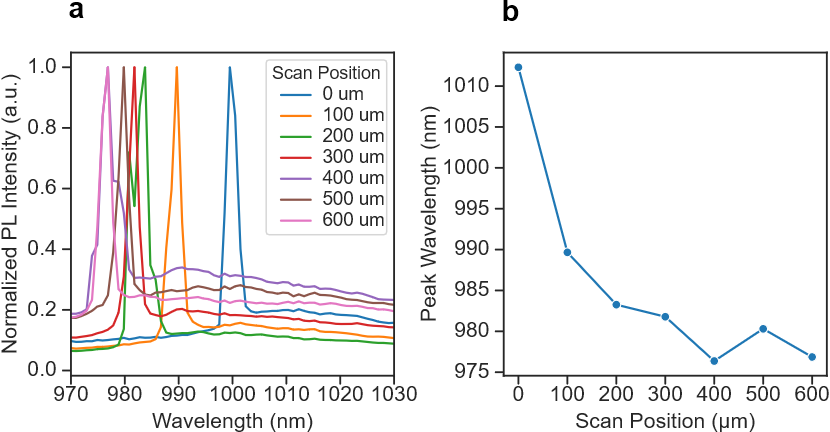

S3.** a) Normalized photoluminescence (PL) intensity for different positions on the sample, taken above the lasing threshold. Each curve represents data acquired at different points starting near the edge of the nano-ridge array and moving inwards. b) The corresponding peak wavelength (nm) at each position (µm), illustrating how the lasing wavelength varies with scan position. Data were collected using a step size of 100 µm.

**Iso-Frequency Contour**

The 3D band structure was simulated using the FDTD (Finite-Difference Time-Domain) method by calculating the resonant modes for each k-vector. A 3D representation of the first TE band is shown in figure S3a,b. The band shows a saddle point at $\Gamma$. Figure S3c shows an iso-frequency contour obtained by taking a plane intersection of the 3D band at the band-edge frequency. This iso-frequency contour matches closely with the results obtained from the experimental backfocal plane image, shown in figure 8e.

**
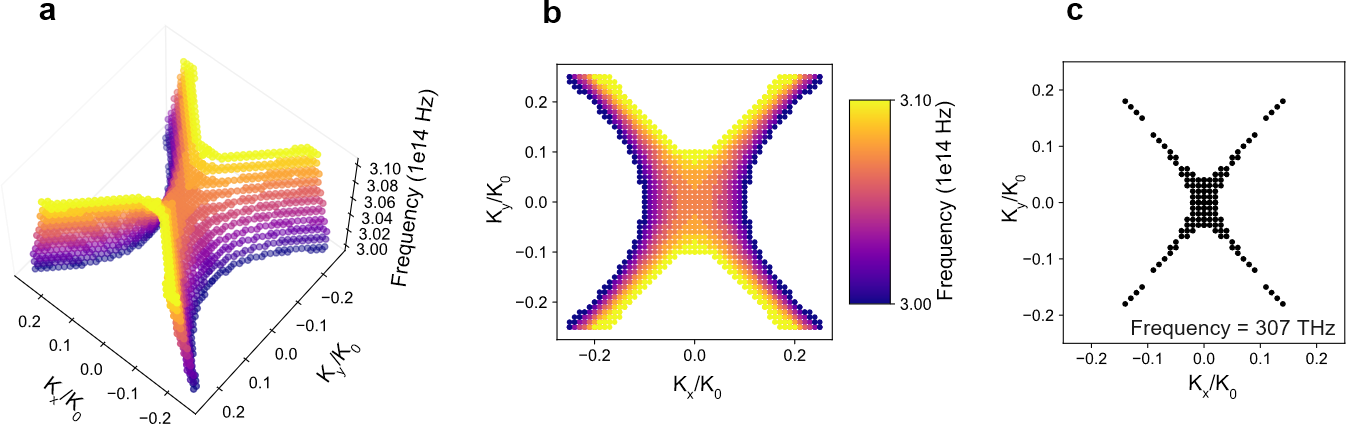
**

**S4.** a) FDTD 3D band surface along k*_x_* and k*_y_* at *TE*_21_*_L_* mode’s resonance frequency. b) Top view of the band surface. c) 2D slice at the saddle point of the surface: 307 THz, showing the iso-frequency contour. The iso-frequency contour matches closely with the results obtained from the back-focal plane experimental images.

**Laser polarization**

We characterised the polarization of an NRSEL with W*_NRSEL_* = 20 µm by passing the emitted light through a polarization analyser and recording the intensity while it was rotated over a range of angles (typically) from 0° (analyser is perpendicular to the nano-ridge) to 180°. The results are shown in figure 4 together with a fitted curve. This shows the device is strongly polarized, with the electrical field mostly aligned with the nano-ridges. The difficulty in perfectly aligning both the sample and the analyser, combined with the fact that the field is not strictly TE-polarized, results in a significant shift of the peak from 90°.


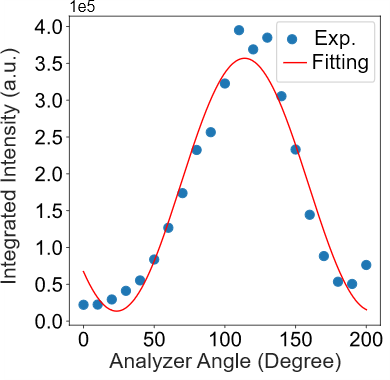


**S5.** The laser light is highly polarized (quasi s-polarized) along the nano-ridge with an extinction ratio of 12.5 dB.
